# Clinical transfer accuracy of pressure-moulded versus 3D-printed drilling guides for orthodontic mini-implants in the anterior palate: a randomized prospective clinical study

**DOI:** 10.1038/s41598-026-50346-z

**Published:** 2026-04-28

**Authors:** Stephan Christian Möhlhenrich, Inas Ayad, Felix Linnerz, Tong Xi, Shankeeth Vinayahalingam, Stefaan Bergé, Gholamreza Danesh, Sachin Chhatwani

**Affiliations:** 1 Department of Dentistry, Section of Orthodontics, Dentofacial Orthopedics and Craniofacial Biology, Radboud University Medical Centre, THK 309, P.O. Box 9101, Nijmegen, 6500 HB The Netherlands; 2 Department of Orthodontics, Witten/Herdecke University, Alfred-Herrhausen Str. 45, 58455 Witten, Germany; 3 Department of Oral and Maxillofacial Surgery, Radboud University Medical Centre, 590, P.O. Box 9101, Nijmegen, 6500 HB The Netherlands

**Keywords:** Orthodontic anchorage procedures, Orthodontic appliances, Temporary anchorage devices, Palate, Orthodontics, Guided implant placement, Diseases, Health care, Medical research

## Abstract

Precise placement of orthodontic mini-implants (OMIs) in the anterior palate, particularly in the median and paramedian regions, is essential for reliable skeletal anchorage. Digitally planned insertion guides may improve transfer accuracy, but clinical data comparing different fabrication workflows remain limited. This randomised clinical trial evaluated the transfer accuracy of pressure-moulded (PM) and three-dimensionally printed (3DP) insertion guides for palatal mini-implant placement in these regions. Thirty patients requiring palatal skeletal anchorage were randomly allocated (1:1) to receive either PM or 3DP insertion guides. Two OMIs were placed per patient, either in the median or paramedian region of the anterior palate. Planned and achieved implant positions were compared after insertion using three-dimensional superimposition. The primary outcome was three-dimensional angular deviation of the implant axis. Secondary outcomes included linear deviations at the implant shoulder and tip and angular deviations. Between-group comparisons were performed using independent-samples t-tests on patient-level averages. No intraoperative complications or missing data occurred. Overall, mean three-dimensional angular deviation was significantly lower for PM than for 3DP guides (3.61 ± 1.29° vs. 5.77 ± 2.45°, *p* = 0.006). Linear deviations were comparable between groups at the implant shoulder and higher at the implant tip, with no statistically significant differences (shoulder: PM 0.81 ± 0.27 mm vs. 3DP 0.84 ± 0.34 mm, *p* = 0.778; tip: PM 1.25 ± 0.40 mm vs. 3DP 1.49 ± 0.58 mm, *p* = 0.199). Both guide types achieved high clinical transfer accuracy; however, PM guides showed lower angular deviations, particularly for paramedian placement. Overall, differences between fabrication workflows were statistically significant but mainly affected angular precision and remained within clinically acceptable ranges.

*Trial Registration*: German Clinical Trials Register (File Number DRKS 00028967, registered on May 04, 2022).

**Supplementary Information:**

The online version contains supplementary material available at 10.1038/s41598-026-50346-z.

## Introduction

Skeletal anchorage has dramatically transformed the management of complex orthodontic cases. Its common use is attributed to its independence from patient compliance and its ability to deliver continuous forces to teeth without causing unwanted side effects. Orthodontic mini-implants (OMIs) are recommended for a range of applications, including the anteroposterior movement of teeth, space closure, molar intrusion, and transverse and sagittal maxillary post-development^1,2^. Optimal insertion areas include the interradicular alveolar bone of the upper and lower jaws, and the anterior palate of the maxilla^3^. The anterior palate, in particular, has gained favor for the placement of OMIs due to the absence of dental roots and adequate bone volume. However, the bone availability in this region varies significantly based on the patient’s age and sex^4,5^. The ideal implant placement is typically medial or paramedial to the palatal suture and near to the third pair of palatal rugae^6^.

Digital planning for the insertion of OMIs helps to minimize risks like root surface damage and implant failure due to inadequate bone quality. Utilizing an insertion guide (IG) in conjunction with digital planning can achieve precise bone insertion depths, even when palatal bone is limited, and ensure parallel placement of multiple OMIs, leading to a more predictable treatment^7–10^.

Computer-assisted surgery (CAS) for mini-implant placement can be performed using different approaches. Non-guided placement corresponds to freehand insertion, whereas guided workflows can be subdivided into static computer-assisted surgery (sCAS), dynamic computer-assisted surgery (dCAS), and robotic computer-assisted surgery (rCAS)^11^. In orthodontics, static systems using prefabricated insertion guides are most commonly applied due to their clinical feasibility and cost-effectiveness.

Digital planning can be based on lateral cephalograms or three-dimensional imaging modalities such as CT or CBCT. The latter can overcome the limitations of two-dimensional (2D) planning, such as unrecognizable images or overlaps^12–14^. An insertion template can be created by superimposing a digital study model using specialized software. In addition, virtually planned guides are typically manufactured using conventional methods, such as thermoforming on working models, or are three-dimensionally fabricated through additive and subtractive processes, like 3D printing or milling^15,16^. Early clinical reports demonstrated that CAD/CAM-based insertion guides enable precise and safe placement of mini-implants in the anterior palate and allow simultaneous appliance delivery in a single appointment^17,18^. Furthermore, these guides are available in various designs, including gingiva- or tooth-supported templates, and can be obtained as commercially customized insertion templates or as in-house planned and fabricated insertion guide^8–10^. Additionally, digital planning allows for the simultaneous insertion of multiple OMIs and the placement of custom-made orthodontic devices in just one appointment^19–21^.

Several preclinical studies have evaluated the accuracy of insertion guides (IGs) for OMI placement. Industrially manufactured guides proved more consistent than in-house versions^22^, and conventional Pattern Resin guides were more precise than 3D-printed ones, which still provided sufficient accuracy^15^. Greater guide extension with tooth support increased transfer accuracy^8,23^, while skeletonized designs showed larger deviations at the implant neck^23^. Although these studies provide valuable methodological insight, most available evidence is derived from preclinical models, such as cadaver heads, typodonts, or in vitro simulations. Consequently, clinical in vivo data under real treatment conditions remain limited. Furthermore, potential differences in transfer accuracy between median and paramedian insertion sites have not been systematically investigated in a controlled clinical setting.

Therefore, the present randomized clinical study aimed to evaluate the in vivo transfer accuracy of two static, digitally planned insertion guides fabricated using different workflows—three-dimensionally printed and conventionally pressure-moulded—for palatal orthodontic mini-implant placement, with particular emphasis on site-specific differences between median and paramedian insertion.

Based on previous experimental findings indicating that insertion guides with increased mechanical support and surface coverage may improve transfer accuracy^8^, a directional hypothesis was formulated. It was assumed that pressure-moulded guides, due to their extended support on the palatal surface, would result in lower transfer deviations compared to three-dimensionally printed guides. In addition, transfer accuracy was expected to vary between median and paramedian insertion sites.

## Materials and methods

This study was conducted as a prospective clinical investigation assessing the transfer accuracy of digitally planned insertion guides for orthodontic mini-implant placement in a randomised two-arm design (PM vs. 3DP). Ethical approval was obtained from the ethics committee of Witten/Herdecke University, Germany (Decision Number 165/2020). The study was conducted in accordance with the Declaration of Helsinki and followed CONSORT recommendations for randomised clinical trials.

### Protocol and study design

The study protocol was registered in the German Clinical Trials Register (DRKS00028967) on 04/05/2022 as a prospective, two-arm, parallel-group randomized clinical trial comparing pressure-moulded and three-dimensionally printed insertion guides for palatal orthodontic mini-implant placement.

### Participants

A total of 30 patients underwent orthodontic mini-implant insertion (mean age 15.9 ± 4.8 years, range 9.9–31.2 years) (Supplementary Table S1). Patients were randomly allocated in a 1:1 ratio to receive either pressure-moulded guides (PM; *N* = 15) or three-dimensionally printed guides (3DP; *N* = 15). Two OMIs were placed per patient. All participants, and legal guardians when applicable, provided written informed consent. No patients were lost to follow-up, as all measurements were obtained immediately after insertion.

### Eligibility criteria

Inclusion criteria for the study are subjects with a need for orthodontic treatment that also requires skeletal anchorage for different orthodontic treatment reasons. Exclusion criteria include patients who cannot be treated due to disabilities of different origin, minors without parental consent, pregnant individuals, those undergoing anticoagulant therapy, individuals with uncontrolled diabetes, acute periodontal disease, nicotine consumption, severe mental disorders, seizure disorders, or previous operations in the insertion area. All eligible patients were recruited consecutively in the outpatient clinic.

### Randomisation and blinding

Participants were randomly allocated in a 1:1 ratio to receive either pressure-moulded or three-dimensionally printed insertion guides using simple randomisation. The random allocation sequence was generated by coin toss by the investigator responsible for participant recruitment prior to guide fabrication. Allocation was performed immediately after sequence generation by the same investigator, without any concealment mechanism. Outcome assessment was conducted by an independent examiner blinded to group allocation.

### Digital planning

Virtual planning was performed using lateral cephalograms (Orthophos SL 2D, Dentsply Sirona, York, PA, USA) and intraoral scans (iTero Element 2, Align Technology, San Jose, CA, USA). ^8,10,14,24^. The planning protocol was specifically intended for palatal mini-implant placement in the median and paramedian region of the anterior palate.

**Fig. 1:**
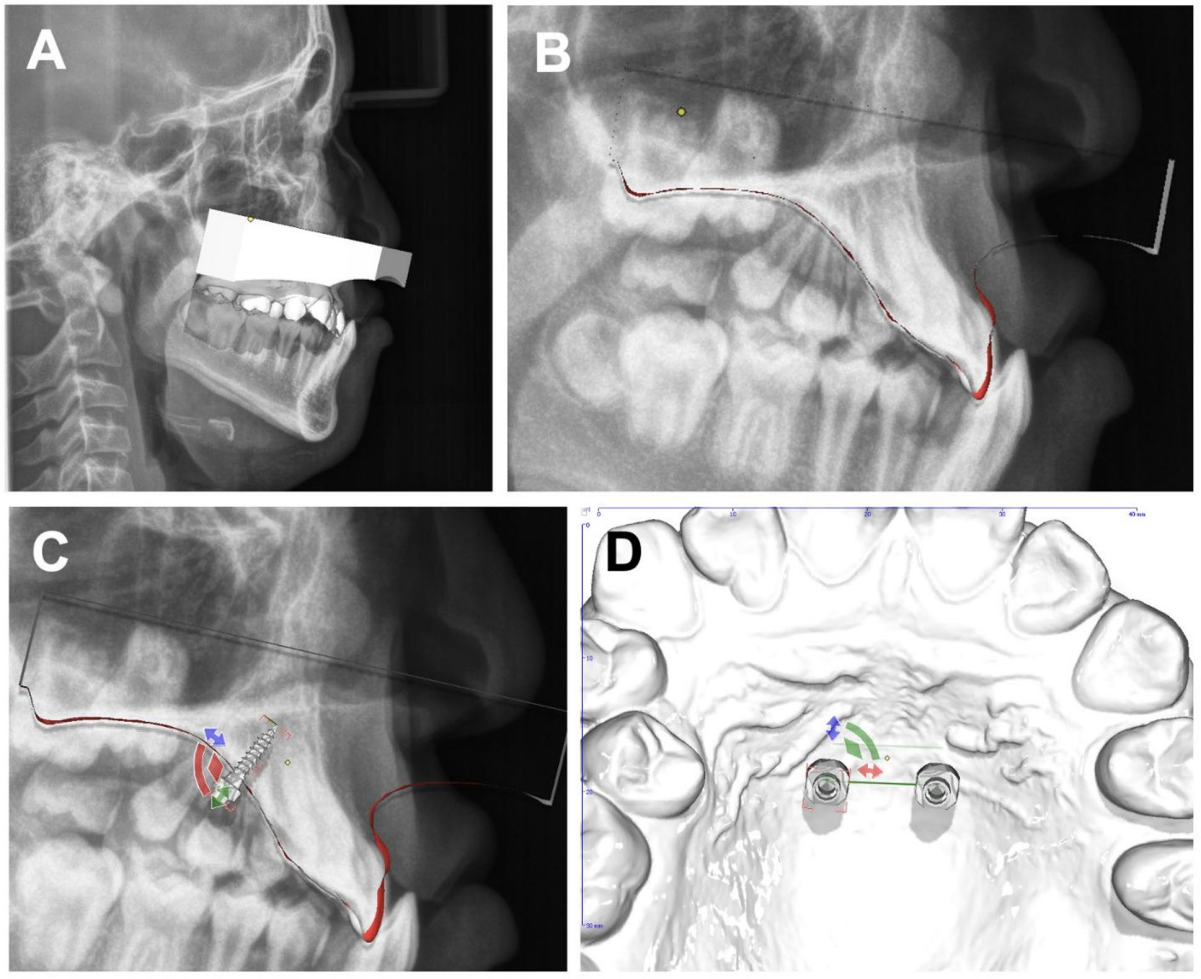
Software-assisted virtual planning of paramedian implantation: (**A**) Superimposed virtual situation model and lateral cephalograms aligned, (**B**) Longitudinal section through the model for visualization of hard tissue structures, (**C**) Fine-tuning of implant position with respect to angulation and length, (**D**) Virtual positioning of implants in the paramedian region.

The intraoral scans were superimposed with the corresponding lateral cephalograms using dedicated software (TADmatch, OnyxCeph, Image Instruments GmbH, Chemnitz, Germany) to combine the three-dimensional dental surface information with the sagittal radiographic assessment of the available palatal bone (Fig. 1A, B). Because the planning dataset was based on fusion of the intraoral scan and the lateral cephalogram, the available anatomical information was limited compared with CBCT-based planning. However, this approach is considered sufficient for mini-implant placement in the median and paramedian region of the anterior palate, where anatomical variability is low. Based on the fused dataset, the implant positions were defined virtually on the digital model. The lateral cephalogram was used for evaluation of the vertical bone supply and for fine adjustment of implant angulation, direction, and length (Fig. 1C, D). All mini-implants (PSM Medical Solutions, Gunningen, Germany) had a diameter of 2.3 mm, with lengths chosen based on individual bone supply, approximately 7, 9, and 11 mm. They were positioned at an angle of approximately 90° to the cortical layer of the hard palate. The distances between the implants ranged from a minimum of 6 mm to a maximum of 12 mm.

### Manufacturing process

**Fig. 2:**
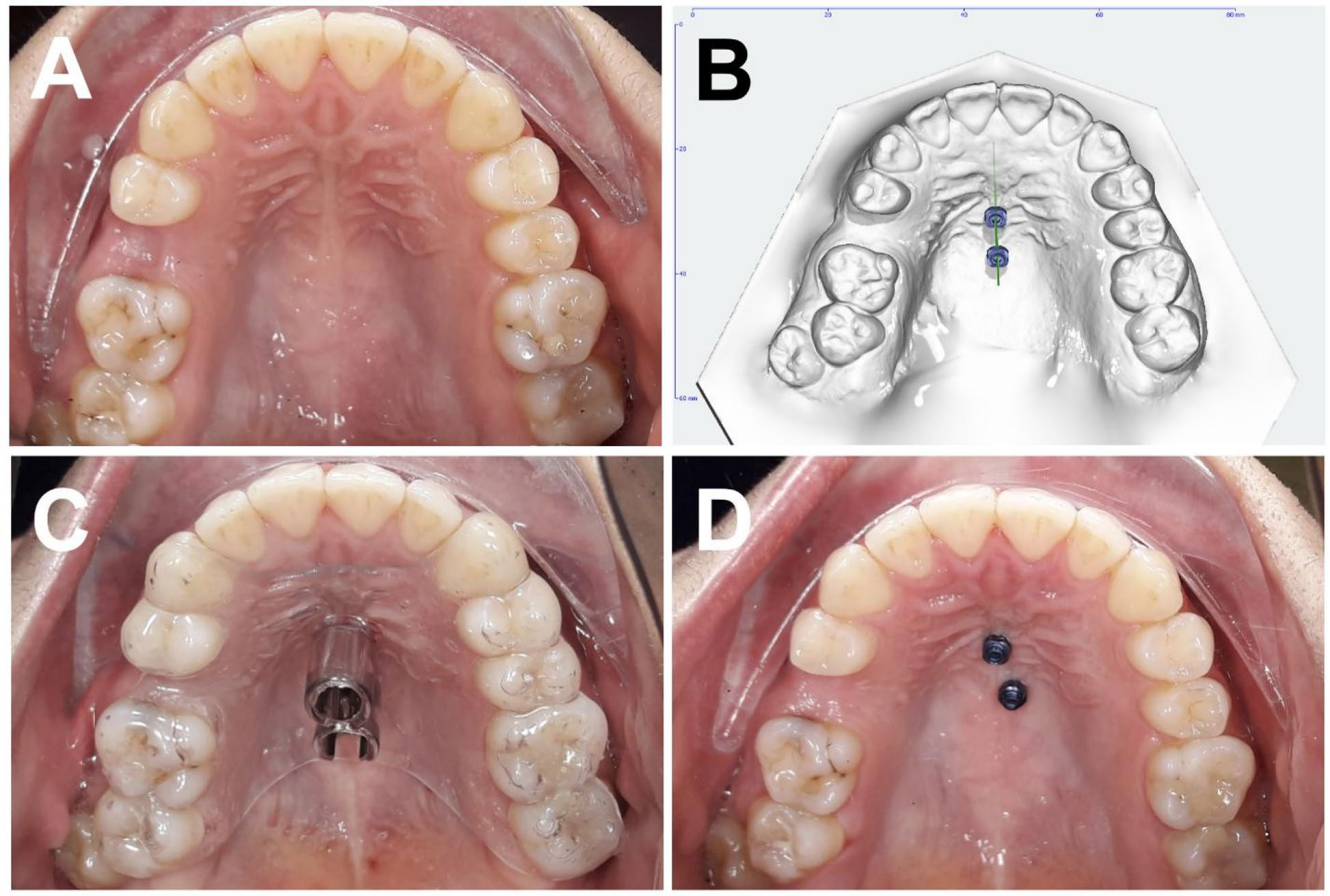
(**A**) Clinical baseline situation, occlusal view of the maxilla before median OMI insertion, (**B**) Virtual median OMI planning, (**C**) Conventional pressure-moulded drilling guide in situ, (**D**) Clinical situation, occlusal view of the maxilla after median OMI insertion.

For manufacturing conventional PM guides, pillars for receiving the corresponding drill sleeves (PSM Medical Solutions, Gunningen, Germany) replaced the mini-implants on the underlying simulation model to construct the final working model, which was finally manufactured by a 3D printer (Form 2, Formlabs, Somerville, Massachusetts, USA). Next, templates were created on the working model. The drill sleeves were positioned on the pillars, and undercuts were minimally blocked out using thermal wax. A spacer foil (SCHEU Dental, Iserlohn, Germany) was then deep-drawn over the model and trimmed around the drill sleeves. Following this, pressure-moulded material (Duran 1.5 mm; Biostar Versatile pressure moulding machine, SCHEU Dental, Iserlohn, Germany) was thermoformed onto the prepared model. Finally, the splint was finished, including the grinding of the area around the drill sleeves to ensure a precise fit (Fig. 2).

**Fig. 3:**
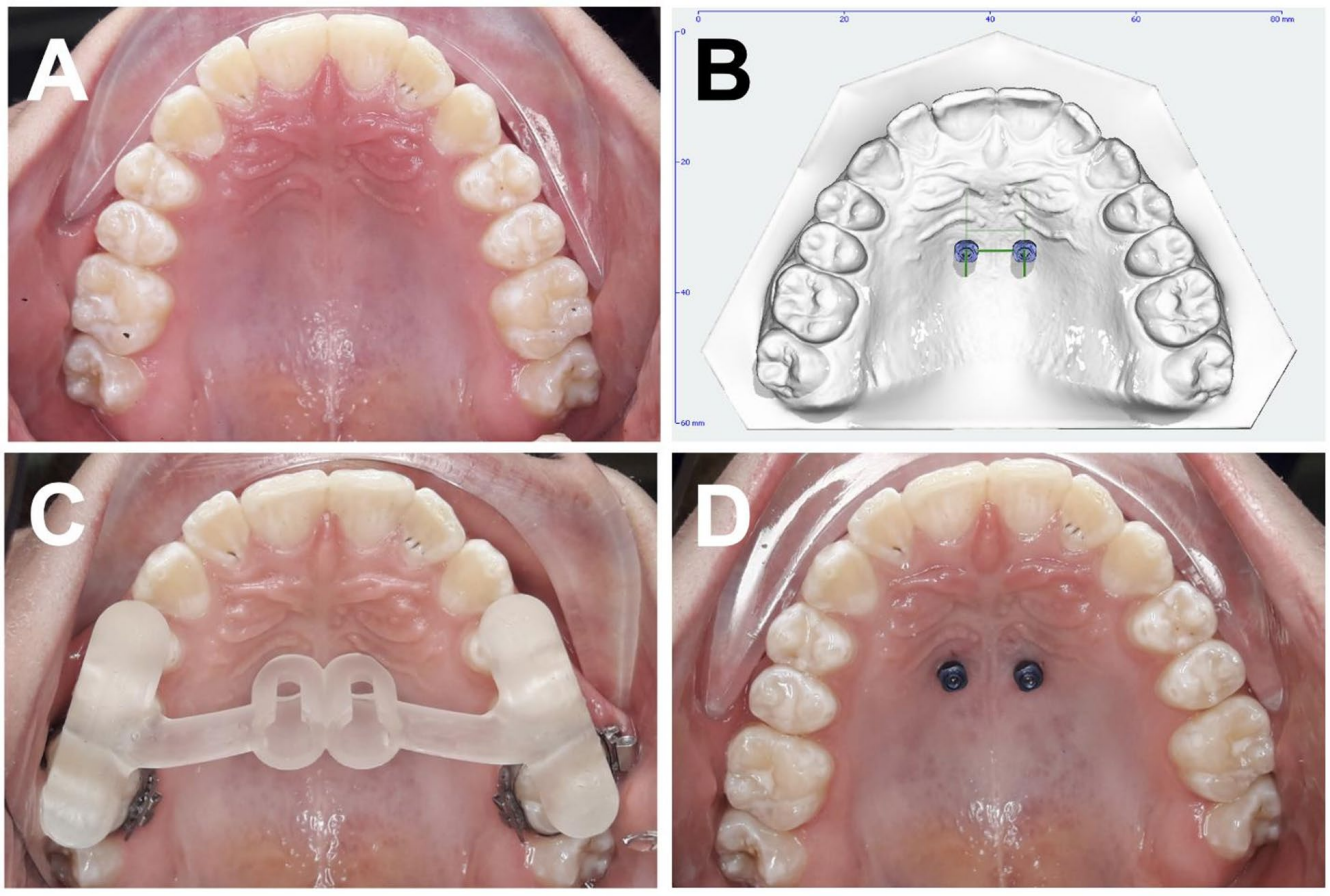
(**A**) Clinical baseline situation, occlusal view of the maxilla before paramedian OMI insertion, (**B**) Virtual paramedian OMI planning, (**C**) 3D-printed drilling guide in situ, (**D**) Clinical situation, occlusal view of the maxilla after paramedian OMI insertion.

For designing the CAD/CAM-based SG (Fig. 3) on the underlying simulation model, the OrthoApps 3D module of OnyxCeph software was used. Two cylindrical guide sockets with a defined length were fittingly arranged around the OMIs and their axes to reproduce the angle of insertion and prevent the OMI from penetrating beyond the intended depth into the bone. The design of the SG was bilaterally extended to the posterior segments and usually comprised 3 teeth on each side and covered them occlusally and buccally to the anatomical equator for support and stabilization. In general, the two premolars and the first molar were used. Afterwards, the resulting SG was then 3D-printed for each patient according to the printing protocol (Surgical Guide Resin; Form 2, Formlabs, Somerville, Massachusetts, USA) (Fig. 3).

### Implant placement

Each patient underwent implant insertion under local anaesthesia. The guides were positioned on the teeth and held in place manually. The implant insertion was performed following predrilling (diameter: 1.8 mm). For the guided OMI placement, the corresponding commercially available drill and screw holder (PSM Medical Solutions, Gunningen, Germany) were used. All guided insertions were performed by experienced practitioners under the supervision of a senior clinician (S.C.M.), following a review of the virtual treatment plan. A contra-angle handpiece drive with a corresponding prosthetic screwdriver (iSD900, NSK Europe GmbH, Eschborn, Germany) was used for predrilling and OMI insertion in both groups. The torque was limited to 40 Ncm with a 25-rpm turning speed. Drilling and insertion automatically stopped when the screwdriver reached vertical stop to ensure the planned insertion depth.

Potential adverse events were defined as material-related complications (e.g., screw fracture), anatomical limitations (e.g., low bone density), or procedural problems (e.g., patient non-cooperation). As the study focused solely on implant insertion, only intraoperative complications were considered relevant. No adverse events occurred during implant placement. All procedures were supervised by a consultant orthodontist (S.C.M. or S.C.), who confirmed the absence of adverse events at the time of insertion.

### Measurement methods

**Fig. 4:**
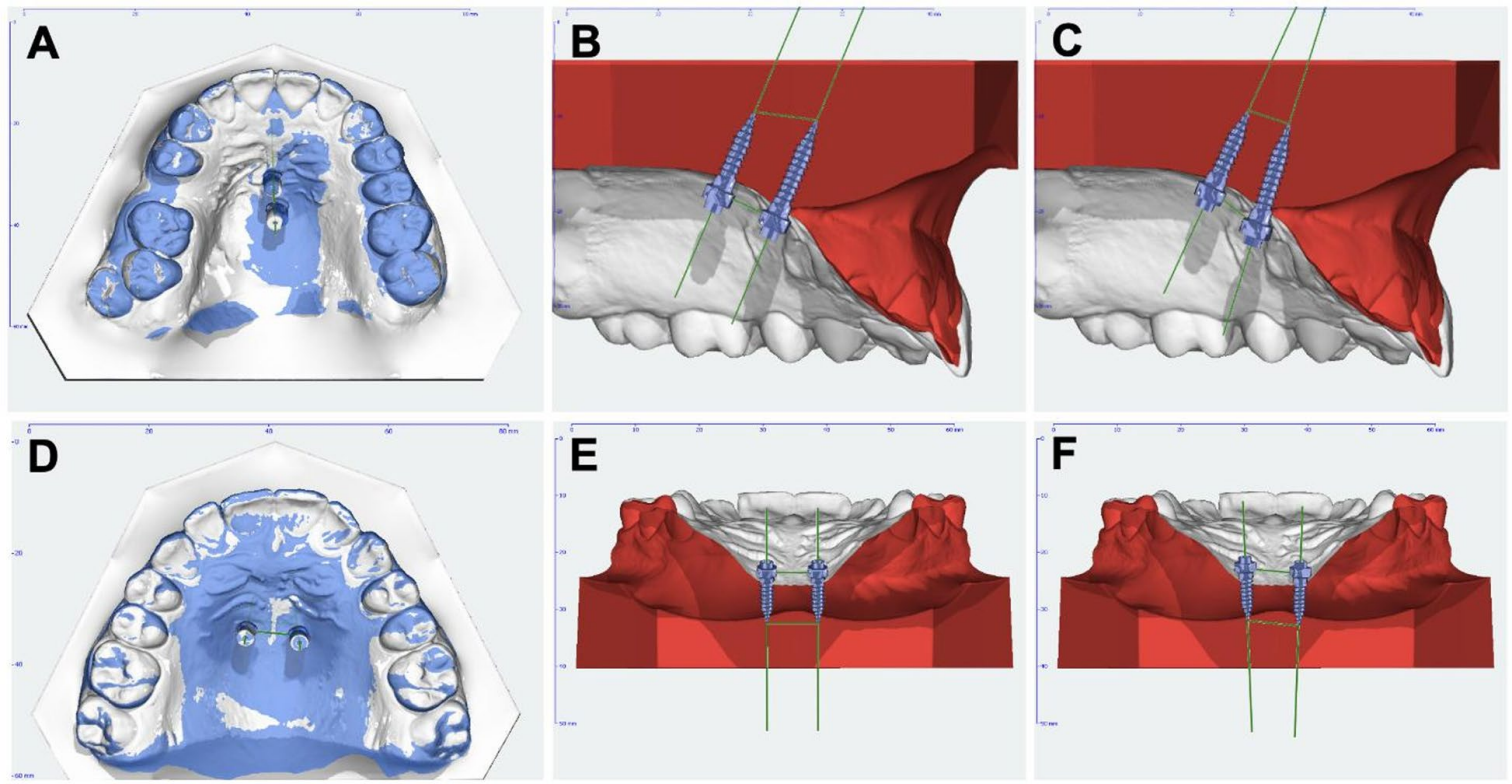
(**A**) Superimposition of the planned (white) and achieved (blue) median OMI positions, (**B**) Alignment of the implants relative to each other within the virtual planning for median placement, (**C**) Reconstructed OMI positions illustrating the resulting final median implant positions, (**D**) Superimposition of the planned (white) and achieved (blue) paramedian OMI positions, (**E**) Alignment of the implants relative to each other within the virtual planning for paramedian placement, (**F**) Reconstructed OMI positions illustrating the resulting final paramedian implant positions.

To record the actual OMI positions, scan bodies (PEEK mini, PSM Medical Solutions, Gunningen, Germany) were placed on the inserted OMIs, followed by an intraoral scan (iTero Element 2, Align Technology, California, USA). The postoperative scan was registered to the virtual planning dataset (T0) by surface-based superimposition of the dental arch and palatal structures, which served as stable reference areas for best-fit alignment (Fig. 4). Because the scan bodies have a predefined geometry, the spatial position and angulation of each inserted OMI (T1) could be reconstructed within the software. Alignment and deviation analysis were performed using OnyxCeph software with a subordinate calculation module in a predefined Cartesian coordinate system. The deviations between T0 and T1 were measured in millimetres at the OMI head and tip levels and calculated along the x-, y-, and z-axes. Three-dimensional linear deviations were calculated as the Euclidean distance between planned and achieved positions:

$$\:\sqrt{\left\{{\left({a}_{1}-\:{b}_{1}\right)}^{2}+\:{\left({a}_{2}-\:{b}_{2}\right)}^{2}+\:{\left({a}_{3}-\:{b}_{3}\right)}^{2}\right\}}$$

In addition, angular deviations of the longitudinal axes were calculated in degrees using the vector angle formula:

$$\cos (\alpha )=\frac{{\overrightarrow a \cdot \overrightarrow b }}{{|\overrightarrow a |\quad |\overrightarrow b |}}$$

Angular deviations were analysed in three-dimensional space and additionally projected onto the YZ (coronal), XY (sagittal), and XZ (transverse) planes to assess direction-specific deviations.

All measurements were performed by a single examiner experienced in digital orthodontic planning who was blinded to group allocation. Because the implant position was defined by the geometry of the scan bodies and calculated automatically within the software, operator-dependent variability was reduced. However, no formal intra- or inter-examiner reproducibility analysis was performed, which should be considered when interpreting the measurement accuracy.

The prespecified primary outcome was the three-dimensional angular deviation (degrees) between the planned and achieved implant axis measured immediately after insertion. Measurements were performed using OnyxCeph. Prespecified secondary outcomes included linear deviations (mm) at implant shoulder and tip as well as plane-specific angular deviations in the sagittal (XY) and coronal (YZ) planes.

### Sample size calculation

The sample size calculation was based on angular deviation data from a published cadaveric study reporting mean deviations of 2.81 ± 2.69° and 6.22 ± 4.26° for different insertion guides^8^. From these values, a pooled standard deviation was obtained, yielding an effect size of d ≈ 0.96 for an independent-samples comparison. Based on the directional hypothesis derived from these data and the assumption that pressure-moulded guides provide greater palatal support extension than three-dimensionally printed guides, resulting in improved transfer accuracy, a one-sided independent-samples t-test was used for sample size estimation. Under assumptions of α = 0.05, and power = 0.80, G*Power (Version 3.1.9.4) indicated a required sample size of 15 patients per group. Accordingly, the final target sample size was set to 15 patients per group, resulting in a total sample size of 30 patients.

### Statistical analysis

The Kolmogorov-Smirnov test for normality was applied to the data. A normal distribution was observed in the study groups and subgroups (median, paramedian placement). Therefore, comparisons between the two independent groups (PM vs. 3DP) were performed using independent-samples t-tests. Because two OMIs were placed per patient, the patient was defined as the unit of analysis. Values from the two implants (right/left for paramedian placement; anterior/posterior for median placement) were averaged per patient prior to statistical analysis, resulting in *n* = 15 per group. Within-patient comparisons (anterior vs. posterior and right vs. left implant positions) were analysed using paired t-tests to account for the dependent structure of the data. All analyses were conducted using Prism (version 10, GraphPad Software Inc., La Jolla, CA, USA). The level of significance was set at *p* ≤ 0.05. All results are expressed as mean ± standard deviation (SD).

## Results

Between November 2022 and May 2025, 116 patients were assessed for eligibility. 86 patients declined participation, of whom 41 provided financial reasons for the mini-implant insertion or the use of guides, and 33 reported pain-related concerns (*n* = 17), discomfort (*n* = 9), or potential speech impairment (*n* = 7) and 12 provided no reason. Thirty patients were randomised to PM (*N* = 15) or 3DP (*N* = 15) and were analysed for the primary outcome after OMI placement (Supplementary Figure S1). All measurements were obtained immediately after insertion, and no missing data occurred or intraoperative complications, including screw fracture or inadequate primary stability, were observed in both groups (PM 0/15; 3DP 0/15). In particular, no screw fractures, failures to reach adequate torque, or patient-related complications occurred.

Baseline demographic characteristics were comparable between groups (Supplementary Table S1). The mean age was 15.9 ± 4.8 years (PM: 15.0 ± 4.9 years; 3DP: 16.7 ± 4.8 years), and sex distribution was balanced between groups.

**Fig. 5:**
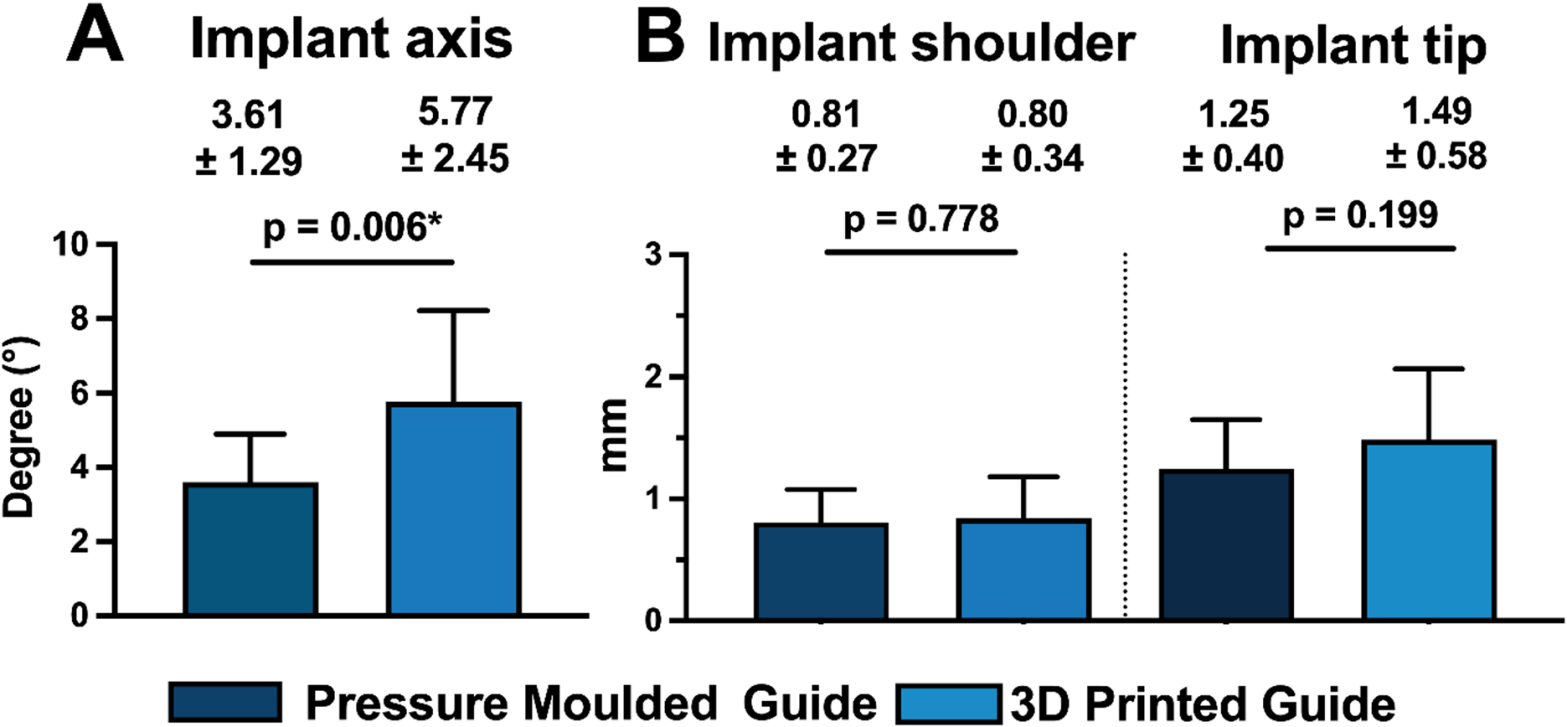
Bar chart illustrating the overall maximum angular deviations of the implant axis in three-dimensional space (**A**), as well as in the coronal (**B**) and sagittal (**C**) planes, compared to the type of insertion guide used (pressure-moulded vs. 3D-printed). Significance levels: * = *p* ≤ 0.05.

Overall implant placement deviations are summarized in and illustrated in Fig. 5. Linear deviations at the implant shoulder and tip were comparable between PM and 3DP guides (shoulder: PM 0.81 ± 0.27 mm, 3DP 0.84 ± 0.34 mm; tip: PM 1.25 ± 0.40 mm, 3DP 1.49 ± 0.58 mm). In contrast, three-dimensional angular deviation of the implant axis was higher in the 3DP group (5.77 ± 2.45°) compared with the PM group (3.61 ± 1.29°). This trend is reflected in Fig. 5, indicating increased angular deviations for 3DP guides (Table 1).

**Table 1:** Implant Placement Deviations According to Insertion Protocol: Means, Standard Deviations, and 95% Confidence Intervals.

Deviation	Insertion protocol	*N*	Mean	SD	Min	Max	95% CI
Implant shoulder	PM	15	0.81	0.27	0.32	1.31	1.02–1.47
	3DP	15	0.84	0.34	0.20	1.50	1.17–1.81
Implant tip	PM	15	1.25	0.40	0.45	1.91	1.00–1.52
	3DP	15	1.49	0.58	0.50	2.75	1.10–1.90
Implant axis (XYZ)	PM	15	3.61	1.29	1.20	6.30	2.89–4.32
	3DP	15	5.77	2.45	1.80	9.80	4.42–7.13

**Fig. 6:**
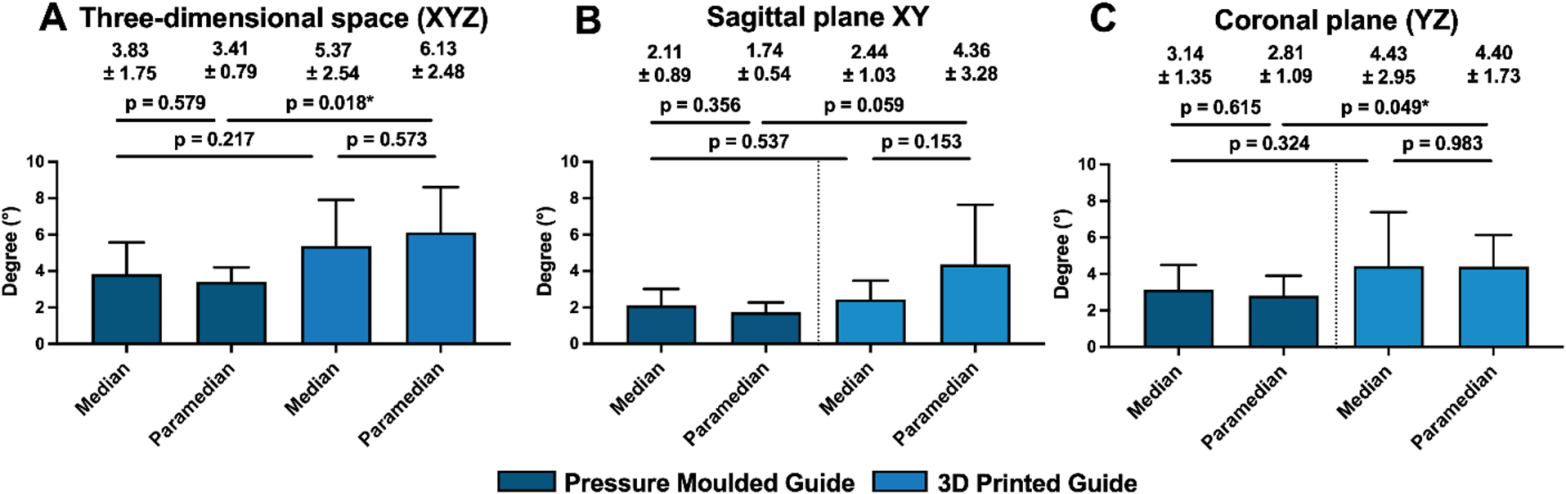
Bar chart illustrating the maximum angular deviations of the implant axis in three-dimensional space (**A**), as well as in the coronal (**B**) and sagittal (**C**) planes, compared to the planned position. Results are shown based on the type of insertion guide used (pressure-moulded vs. 3D-printed) and implant position (median vs. paramedian). Significance levels: * = *p* ≤ 0.05.

The three-dimensional (XYZ) and plane-specific angular deviations of the implant axis between the planned and achieved positions in the sagittal (XY) and coronal (YZ) planes are summarized in Table 2 and illustrated in Fig. 6. For median placement, mean three-dimensional angular deviation was lower in the PM group (3.83 ± 1.75°) compared with the 3DP group (5.37 ± 2.54°) but not statistically significant (*p* = 0.217). In contrast, for paramedian placement, angular deviation was statistically significantly lower in the PM than in the 3DP group (PM: 3.41 ± 0.79°, 3DP: 6.13 ± 2.48°, *p* = 0.018). Accordingly, across both planes, PM guides demonstrated lower angular deviations than 3DP guides for paramedian placements. The magnitude of differences varied by plane, with the largest discrepancies observed for paramedian placement in the sagittal (XY) plane (PM: 1.74 ± 0.54°, 3DP: 4.36 ± 3.28°, *p* = 0.059) compared to the coronal (YZ) plane (PM: 2.81 ± 1.09°, 3DP: 4.40 ± 1.73°, *p* = 0.049).

**Table 2:** Implant Placement Deviations According to Insertion Protocol and Position: Means Standard Deviations and 95% Confidence Intervals.

Deviation	Insertion protocol	Placement	*N*	Mean	SD	Min	Max	95% CI
Implant shoulder	PM	Median	7	0.84	0.35	0.32	1.31	0.51–1.16
		Paramedian	8	0.78	0.20	0.50	1.15	0.62–0.95
	3DP	Median	7	0.71	0.37	0.20	1.30	0.37–1.06
		Paramedian	8	0.95	0.29	0.55	1.50	0.71–1.19
Implant tip	PM	Median	7	1.29	0.52	0.45	1.91	0.81–1.77
		Paramedian	8	1.21	0.30	0.80	1.81	0.96–1.46
	3DP	Median	7	1.36	0.44	0.70	1.95	0.95–1.77
		Paramedian	8	1.60	0.68	0.50	2.75	1.03–2.17
Implant axis (XYZ)	PM	Median	7	3.83	1.75	1.20	6.30	2.21–5.45
		Paramedian	8	3.41	0.79	2.30	4.90	2.75–4.07
	3DP	Median	7	5.37	2.54	1.80	8.90	3.02–7.72
		Paramedian	8	6.13	2.48	2.20	9.80	4.05–8.20
Implant axis / Sagittal plane (XY)	PM	Median	7	2.11	0.90	0.80	3.50	1.29–2.94
		Paramedian	8	1.74	0.54	1.00	2.80	1.29–2.19
	3DP	Median	7	2.44	1.03	1.20	4.10	1.49–3.40
		Paramedian	8	4.36	3.28	0.70	10.0	1.62–7.10
Implant axis / Coronal plane (YZ)	PM	Median	7	3.14	1.35	1.20	5.10	1.89–4.39
		Paramedian	8	2.81	1.09	1.30	4.80	1.90–3.72
	3DP	Median	7	4.43	4.43	0.50	8.80	1.70–7.16
		Paramedian	8	4.40	1.73	1.20	6.90	2.95–5.85

PM: Pressure-moulded-formed Guide. 3DP: 3D Printed Guide. FH: Freehand insertion. CI: Confidence interval.

**Fig. 7:**
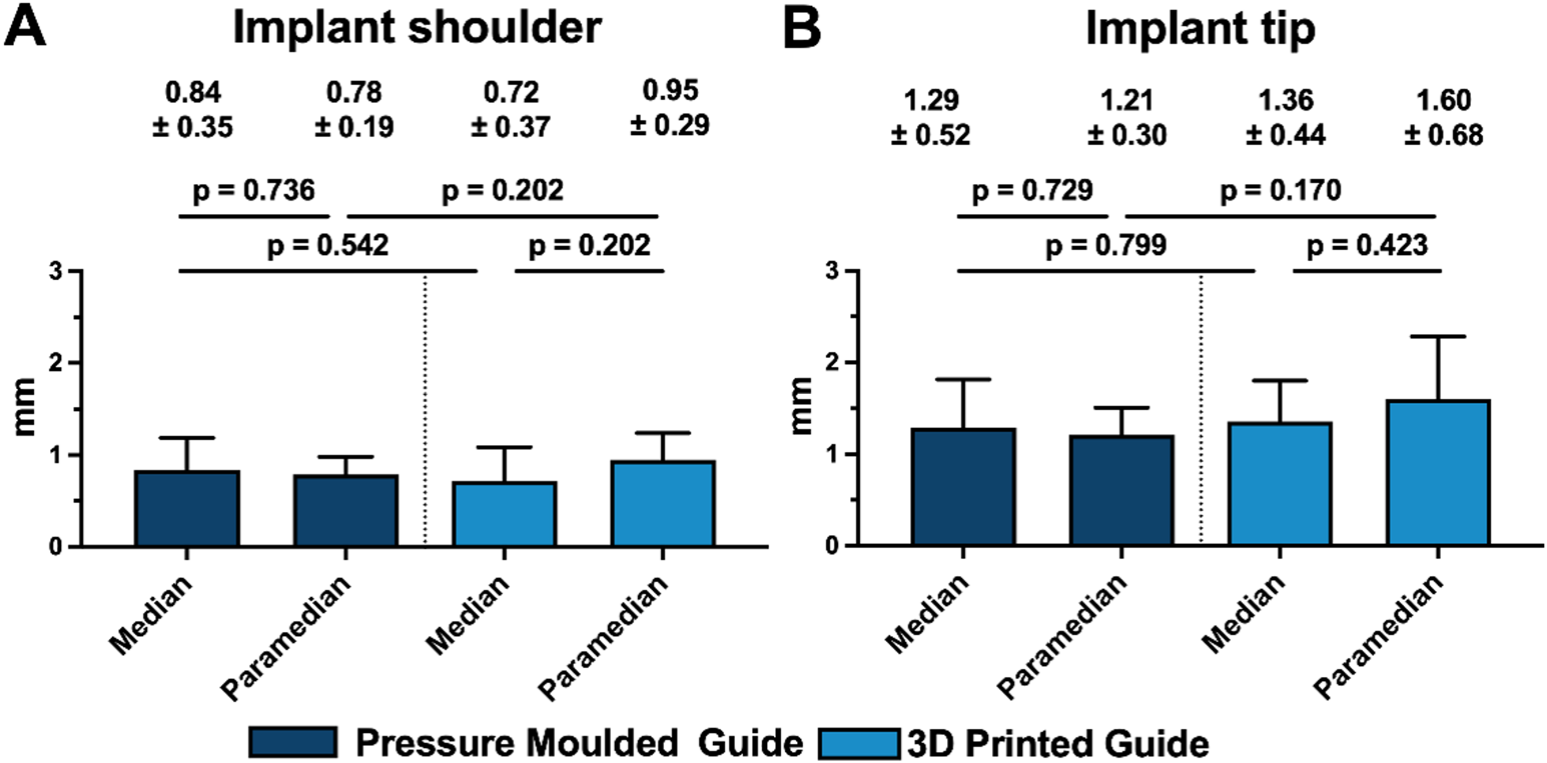
Bar chart illustrating the maximum linear deviations of the achieved implant position compared to the planned position at the implant tip and shoulder. The results are presented based on the type of insertion guide used (pressure-moulded vs. 3D-printed) and the implant position (median vs. paramedian). Significance levels: * = *p* ≤ 0.05.

Linear deviations at the implant shoulder and tip are reported in Table 2 and shown in Fig. 7.

At the implant shoulder, linear deviations were in the submillimetre range for both guide types, with mean values ranging from 0.71 ± 0.37 mm to 0.95 ± 0.29 mm across median and paramedian placements. At the implant tip, deviations were higher than at the shoulder, with mean values ranging from 1.21 ± 0.29 mm to 1.67 ± 0.63 mm across placements. For both measurement points, no statistically significant differences were detected between the groups.

Within-group comparisons of implant position (anterior vs. posterior for median placement; right vs. left for paramedian placement) are summarised in Table 3.

**Table 3:** Linear deviations at the implant tip and shoulder, as well as angular deviations of the implant axis in three-dimensional space (XYZ), and in the sagittal (XY) and coronal (YZ) planes, analysed for each implant based on the type of placement. Statistical comparisons were performed between the two implants within each group (median: anterior vs. posterior; paramedian: right vs. left).

Deviation	Insertion protocol	Median (N = 7)				*p*-Value	Paramedian (N =8				*p*-Value
		Anterior		Posterior			Right		Left		
		Mean ± SD	Range	Mean ± SD	Range		Mean ± SD	Range	Mean ± SD	Range	
Implant tip	PM	1.12 ± 0.47	0.36–1.65	1.47 ± 0.57	0.54–2.17	< 0.001*	1.03 ± 0.229	0.68–1.45	1.39 ± 0.37	0.92–2.17	< 0.001*
	3DP	1.19 ± 0.39	0.62–1.70	1.53 ± 0.49	0.79–2.21	< 0.001*	2.12 ± 0.87	0.71–3.57	1.09 ± 0.49	0.29–1.93	< 0.001*
Implant shoulder	PM	0.61 ± 0.28	0.21–0.97	1.06 ± 0.42	0.43–1.65	< 0.001*	0.76 ± 0.19	0.49–1.12	0.80 ± 0.20	0.51–1.18	< 0.001*
	3DP	0.65 ± 0.35	0.17–1.20	0.78 ± 0.40	0.23–1.40	< 0.001*	1.02 ± 0.31	0.58–1.61	0.88 ± 0.27	0.52–1.39	< 0.001*
XYZ axis	PM	4.15 ± 1.85	1.36–6.78	3.51 ± 1.65	1.04–5.82	< 0.001*	3.07 ± 0.67	2.16–4.35	3.76 ± 0.92	2.44–5.45	< 0.001*
	3DP	4.74 ± 2.35	1.48–7.95	6.01 ± 2.74	2.12–9.85	< 0.001*	7.95 ± 3.15	2.93–12.7	4.31 ± 1.82	1.47–6.89	< 0.001*
YZ axis	PM	3.22 ± 1.38	1.24–5.21	3.07 ± 1.33	1.16–4.99	< 0.001*	2.46 ± 0.960	1.16–4.24	3.16 ± 1.21	1.44–5.36	< 0.001*
	3DP	3.60 ± 2.70	0.09–7.56	5.25 ± 3.21	0.91–10.0	< 0.001*	4.91 ± 1.92	1.40–7.71	3.90 ± 1.55	1.00–6.09	< 0.001*
XY axis	PM	2.18 ± 0.92	0.83–3.60	2.05 ± 0.87	0.77–3.40	< 0.001*	1.86 ± 0.58	1.05–2.99	1.62 ± 0.50	0.95–2.61	< 0.001*
	3DP	1.80 ± 0.84	0.88–3.14	3.08 ± 1.23	1.52–5.06	< 0.001*	6.78 ± 4.52	1.30–14.1	1.94 ± 2.08	0.10–5.94	< 0.001*

PM: Pressure-moulded-formed Guide, 3DP: 3D Printed Guide, *: statistically significant; Significance level: *p* ≤ 0.05.

In the PM group, statistically significant differences between positions were observed for several parameters. For median placement, posterior implants showed higher linear deviations at the implant tip (1.47 ± 0.57 mm vs. 1.12 ± 0.47 mm) and shoulder (1.06 ± 0.42 mm vs. 0.61 ± 0.28 mm) compared to anterior implants (both *p* < 0.001). For paramedian placement, differences between right and left sides were also statistically significant for all parameters, with slightly higher values on the left side for most measurements (all *p* < 0.001).

In the 3DP group, statistically significant positional differences were observed for all parameters (all *p* < 0.001). For median placement, posterior implants showed higher deviations than anterior implants for both linear and angular measurements (e.g., implant tip: 1.53 ± 0.49 mm vs. 1.19 ± 0.39 mm; XYZ axis: 6.01 ± 2.74° vs. 4.74 ± 2.35°). For paramedian placement, marked side-related differences were observed, with higher deviations on the right side, particularly for angular parameters (e.g., XYZ axis: 7.95 ± 3.15° vs. 4.31 ± 1.82°; XY axis: 6.78 ± 4.52° vs. 1.94 ± 2.08°; all *p* < 0.001).

## Discussion

This randomized clinical study demonstrated that both pressure-moulded and three-dimensionally printed guides achieved high transfer accuracy for palatal mini-implant placement. However, pressure-moulded guides showed lower angular deviations, particularly for paramedian insertion, indicating a modest but consistent advantage in angular precision. Linear deviations were comparable between groups and remained within clinically acceptable ranges. In both guide groups, mean linear deviations in implant position remained within 1 mm at shoulder and tip and angular deviations remained below 6.5°, values generally considered clinically acceptable. These findings align with previous studies reporting high transfer precision for template-based techniques. For instance, Augustinowitz et al. demonstrated that digital planning using lateral cephalograms or CBCT achieves angular deviations as low as 3.7°, with tip deviations below 0.6 mm in cadaver heads^24^. Likewise, Schwärzler et al. observed comparable angular accuracy for both 3D-printed and milled insertion guides, with deviations generally under 4° and spatial deviations at the apex around 1 mm^16^. The current study corroborates these values under real-world clinical conditions, providing reassurance that both conventional and digital workflows can achieve high positional accuracy.

While the anterior palate is generally associated with a low risk of direct root damage due to the absence of adjacent dental roots, implant positioning is still constrained by the available bone and adjacent anatomical structures^25,26^. The present results demonstrated lower angular deviations for pressure-moulded guides, particularly for paramedian placement, indicating a statistically significant but quantitatively modest difference between the investigated workflows. Therefore, even small angular deviations may influence the insertion trajectory, available bone support, and the fit of prefabricated orthodontic appliances^25–27^. However, the magnitude of these differences remained within ranges generally considered clinically acceptable, suggesting that their impact on overall clinical outcomes is likely limited under the investigated conditions.

Interestingly, no statistically significant differences between PM and 3DP guides were observed for most linear parameters, and differences in angular deviation were most pronounced for paramedian placement.

Overall, this suggests that, provided the guide design is stable and the clinical fit is accurate, the fabrication method — pressure moulding or 3D printing — has a limited influence on linear transfer accuracy, whereas angular differences may become relevant in anatomically or ergonomically more demanding situations. However, it must be considered that the two investigated workflows differed not only in fabrication method but also in guide design, extension, and support characteristics. Therefore, the observed differences may reflect the combined effect of these workflow-specific factors rather than the manufacturing technique alone. This is consistent with findings from orthodontic indirect bonding workflows, where both thermoformed and 3D-printed transfer trays achieve clinically acceptable accuracy, but tray design and material properties can influence specific dimensions of transfer precision^28,29^. These observations also aligns with Schwärzler et al., who reported no significant difference between milled and printed templates for palatal TADs^16^. The selection of fabrication method may, therefore, be guided more by logistical and economic considerations than by expected clinical outcomes.

The present results are also consistent with findings from Pliska et al., who reported linear deviations of 1.2–2.1 mm at the shoulder and 1.7–2.8 mm at the tip in cadaver models using skeletonized or full-arch 3D-printed guides^23^, whereas in the present study, deviations were lower at the implant shoulder (approximately 0.7–1.0 mm) and within a comparable range at the tip (approximately 1.2–1.6 mm), indicating similar or slightly improved transfer accuracy under clinical conditions. However, they observed maximum angular deviations above 30°, particularly with skeletonized guide designs and pre-drilling, and noted challenges in guide stability and reproducibility depending on occlusal morphology. These findings echo the side-related differences observed in the 3DP group for paramedian placement, suggesting that ergonomic and anatomical factors may contribute to insertion variability in addition to guide fabrication.

In terms of manufacturing methods, Mang de la Rosa et al. showed in a structured in vitro study that conventionally manufactured guides made from Pattern Resin LS (Low Shrinkage) achieved the highest accuracy, outperforming all 3D-printed variants evaluated^15^. They reported median angular deviations below 2° for conventional guides, and up to 6° for some printed templates.

In the present study, linear deviations were comparable between groups (approximately 0.8 mm at the implant shoulder and 1.3–1.5 mm at the tip), whereas angular deviations were lower for pressure-moulded guides (3.6° vs. 5.8°), indicating a measurable advantage in angular precision. However, in contrast to the standardized guide design used in their study, the present workflows differed not only in manufacturing method but also in design, extension, and support characteristics, which may additionally influence transfer accuracy.

Interestingly, the differences in their study were partly attributed to material properties such as flexibility or printing accuracy, which may influence sleeve stability and tolerance. This is consistent with the present findings and suggests that material-related differences may contribute to transfer accuracy, but are likely secondary to factors such as guide design, support, and clinical fit.

In line with these findings, despite comparable linear accuracy between PM and 3DP templates, minor differences emerged. For paramedian placement, PM templates showed higher angular precision, which may relate to differences in guide rigidity and seating behaviour. In contrast, side-related differences were observed in both groups, particularly for paramedian placement. However, these differences were more pronounced in the 3DP group, especially for angular parameters. Despite statistical significance, the magnitude of these differences remained limited, suggesting low clinical relevance. These findings may reflect systematic influences such as insertion direction, operator handedness, or minor variations in guide seating, rather than purely workflow-specific limitations. These observations are consistent with previous findings showing that outcomes depend on both guide design and operator experience^23^.

Practically, both guide types can be integrated into streamlined workflows. The use of guided insertion allows for predictable OMI positioning and paves the way for prefabricated appliance protocols, potentially reducing treatment time and improving patient comfort. While 3DP guides offer compatibility with fully digital processes, PM templates remain a cost-effective and readily accessible alternative, particularly in settings without in-house CAD/CAM facilities.

Looking ahead, further in vivo studies with larger sample sizes and long-term follow-up are needed to assess whether the observed differences in transfer accuracy result in clinically meaningful outcomes. In particular, the integration of digital planning, guided insertion, and one-step appliance delivery remains a promising but still evolving workflow. Comparative studies focusing on time efficiency, cost-effectiveness, and patient-centered outcomes will be critical for guiding evidence-based clinical decisions.

This study has several limitations. First, complete blinding was not possible, since the type of insertion workflow was evident to the operator; however, outcome assessment was performed by an examiner blinded to group allocation. Second, the sample size was relatively small, which may limit the detection of subtle differences between guide types and the generalizability of results. Moreover, as the calculation was based on an effect size derived from cadaveric data, smaller but clinically relevant differences may have remained undetected. Third, the wide age range may have introduced variability in bone characteristics and insertion conditions, although mean ages were comparable between groups. In this context, a split-mouth design might have allowed for better control of inter-individual anatomical variability and increased statistical efficiency; however, a parallel-group design was chosen to reflect clinical workflows and to avoid potential interactions between different guide types within the same patient. Fourth, implant planning was based on the superimposition of lateral cephalograms and intraoral scans rather than CBCT data. While this approach reflects a pragmatic and radiation-reducing clinical workflow, it does not allow for full three-dimensional assessment of root proximity and bone availability, which may limit the precision of anatomical risk evaluation, it provides limited three-dimensional anatomical information compared with CBCT-based planning. This may affect the accuracy of angular and linear planning in relation to surrounding anatomical structures.

Fifth, the study focused exclusively on intraoperative transfer accuracy, and no conclusions can be drawn regarding long-term stability or clinical success of the mini-implants. Finally, randomisation was performed by coin toss without a formal allocation concealment procedure, which may increase the risk of selection bias; however, consecutive recruitment and comparable baseline characteristics suggest that the likelihood of relevant allocation imbalance is low.

## Conclusion

Both pressure-moulded and three-dimensionally printed guides demonstrated high transfer accuracy for palatal mini-implant placement under clinical conditions. Linear deviations were comparable between groups, whereas pressure-moulded guides consistently showed lower angular deviations, particularly for paramedian placement. Although these differences were statistically significant, their magnitude was small and is unlikely to be clinically relevant. Therefore, both workflows can be considered suitable for clinical application, while minor advantages in angular precision may favour pressure-moulded guides in specific situations.

## Supplementary Information

Below is the link to the electronic supplementary material.

Supplementary Material 1

Supplementary Material 2

## Data Availability

The datasets generated and/or analysed during the current study are available from the corresponding author on reasonable request.
