# Supplementary material for: Clinical transfer accuracy of pressure-moulded versus 3D-printed drilling guides for orthodontic mini-implants in the anterior palate: a randomized prospective clinical study
Source: Sci Rep. 2026 Apr 28;16:13626. doi: 10.1038/s41598-026-50346-z (PMC13125631; doi:10.1038/s41598-026-50346-z)
Supplement: Supplementary file 1 — Supplementary Material 1 [file 41598_2026_50346_MOESM1_ESM.docx]

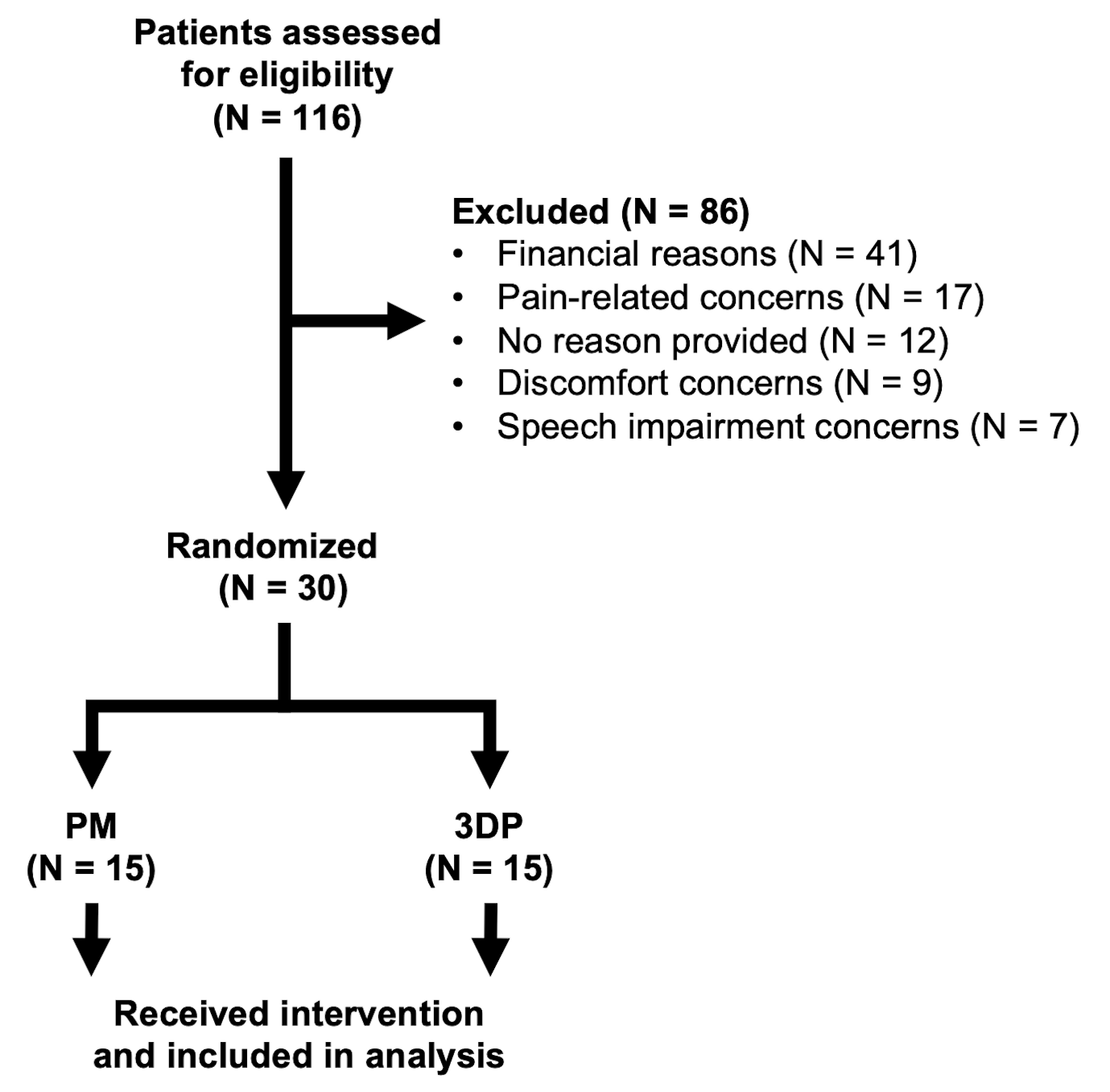


Figure S1: CONSORT flow diagram of patient enrolment, exclusions, randomisation, and allocation to study arms
