# Supplementary material for: Clinical transfer accuracy of pressure-moulded versus 3D-printed drilling guides for orthodontic mini-implants in the anterior palate: a randomized prospective clinical study
Source: Sci Rep. 2026 Apr 28;16:13626. doi: 10.1038/s41598-026-50346-z (PMC13125631; doi:10.1038/s41598-026-50346-z)
Supplement: Supplementary file 2 — Supplementary Material 2 [file 41598_2026_50346_MOESM2_ESM.pdf]

Table S1: Baseline characteristics of the study participants. Values are presented as mean ± standard deviation (SD) for continuous variables and as absolute numbers with percentages for categorical variables. PM = pressure-moulded guide; 3DP = 3D-printed guide.

**Characteristic**	**PM (N = 15)**	**3DP (N = 15)**	**Total (N = 30)**
Age (years), mean ± SD	15.0 ± 4.9	16.7 ± 4.8	15.9 ± 4.8
Sex (female/male)	6/9	9 / 6	15 / 15
